# Spatial Distribution and Health Risk Assessment of Soil Pollution by Heavy Metals in Ijebu-Ode, Nigeria

**DOI:** 10.5696/2156-9614-9.22.190601

**Published:** 2019-05-20

**Authors:** Oludare Hakeem Adedeji, Oluwafunmilayo O. Olayinka, Opeyemi O. Tope-Ajayi

**Affiliations:** Department of Environmental Management and Toxicology, Federal University of Agriculture, Abeokuta, Nigeria

**Keywords:** geographic information system, GIS, heavy metal, health risk, toxicity, urban soils

## Abstract

**Background.:**

Soil pollution by heavy metals in urban areas is of major concern to city planners and policy makers because of the potential threat to human health. Hence, an investigation of soil pollution is crucial to urban environmental assessment and management.

**Objective.:**

To determine the spatial distribution and health risk assessment of seven heavy metals (cadmium (Cd), chromium (Cr), copper (Cu), manganese (Mn), nickel (Ni), lead (Pb), and zinc (Zn)) around Ijebu-ode, southwest Nigeria.

**Methods.:**

Surface soil samples were analyzed for Cd, Cr, Cu, Mn, Ni, Pb, and Zn levels using standard procedures. Geographic information system (GIS) data, pollution indices (enrichment factor, geo-accumulation index), and the health risk assessment model, respectively, were used to analyze the spatial distribution, pollution level, and potential health risk of heavy metals.

**Results.:**

Low pH was observed in the urban soils. The average concentrations of the seven heavy metals investigated were in order of Zn > Pb > Mn > Cu > Cd > Ni > Cr. There was high spatial variation in the distribution patterns of the heavy metals. The cancer risks for Cu, Mn, Pb, and Zn for children (1.50 × 10^−3^ – 2.71 × 10^−2^) and Mn, Pb, and Zn for adults (7.89 × 10^−4^ – 2.97 × 10^−3^) were higher than the acceptable range of 1 × 10^−6^ - 1 × 10^−4^.

**Conclusions.:**

Anthropogenic activities from different urban land uses contribute to the pollution levels and spatial distribution of heavy metals in soils. Increasing pollution of urban soil may contribute to the occurrence of some health risk for residents in the study area.

**Competing Interests.:**

The authors declare no competing financial interests.

## Introduction

Urban centers worldwide are experiencing rapid expansion into surrounding areas. By 2050, about two-thirds of the world's 6.3 billion people are expected to live in cities.^1^ Rapid growth in urban populations is often accompanied by increasing anthropogenic activities that have negative impacts on the urban environment.^2^ Urban soils generally exhibit high spatial heterogeneity due to the complex mixture of both organic and inorganic pollutants, such as heavy metals, compared to soils in rural areas. Heavy metals accumulate in soils from different anthropogenic sources, including inputs from incineration at dumpsites, emissions, and discharges from industrial plants, artisan workshops, and road traffic emissions.^3^,^4^ Concentrations of heavy metals such as lead (Pb), zinc (Zn), cadmium (Cd), chromium (Cr), and copper (Cu) in urban soils vary significantly based on city size, land use type, population density, and traffic volume.^5^,^6^ Heavy metals are of concern due to their persistence, toxicity, nondegradability, and long biological halflives for elimination from the body.^7^,^8^ These toxic metals can be ingested or inhaled from soil and could present human health risks, especially in children.^9–12^ They are the leading cause of a number of health problems such as central nervous disorders, prostate and lung tumors, bone marrow hyperplasia, cardiac hypertrophy, and illnesses of anemia and osteomalacia, among others.^13^,^14^

To safeguard human health and ensure ecosystem integrity, it is important to monitor the pollution levels of heavy metals in urban soils.^15^,^16^ Several approaches, including multivariate statistical methods, geostatistical methods, and geographic information systems (GIS) have been successfully used to identify heavy metal pollutants and the effects of different land uses on their accumulation in urban soil.^16–18^

The present study examined the spatial distribution and levels of heavy metals pollution and estimated the human health risk based on the concentrations of heavy metals (Cd, Cr, Cu, manganese (Mn), nickel (Ni), Pb, and Zn) in urban soil samples from Ijebu-ode, Nigeria, using GIS and multivariate statistics. The study also compared measured median concentrations of these metals with existing published guidelines.

There is a scarcity of such studies in low- and middle-incomes countries like Nigeria, especially studies that examine potential health impact of contamination.

## Methods

**Figure 1:**
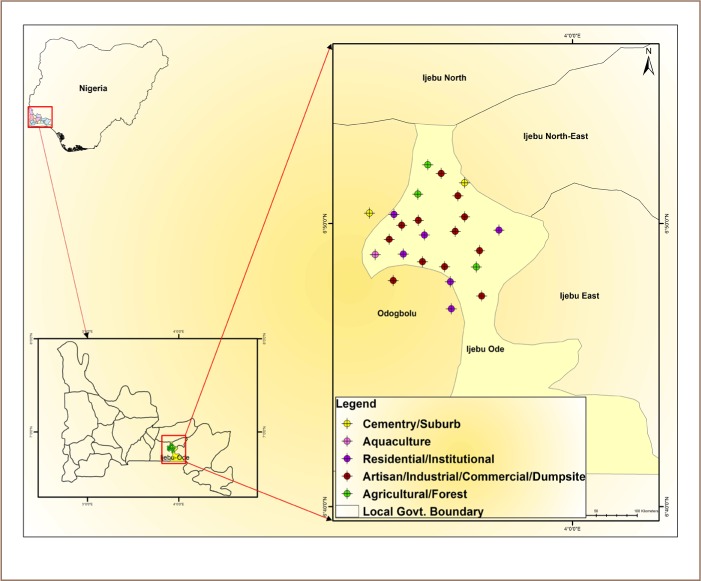
Map of study area showing sampling locations

The present study was conducted in Ijebu–ode city, the second largest city in Ogun State, southwest Nigeria, covering about 192 km^2^ of land. It is located between latitudes 6° 42′ N and 6° 54′ N and longitude 3° 55′ E and 4° 6′ E (*Figure 1*) and has an average elevation of 120 m. It is situated about 110 km by road northeast of Lagos, the capital of Nigeria. It had an estimated population of 222 653 as of 2006, and a population density of 481 persons per hectare.

The climate falls into two distinct seasons, the Harmattan (dry) season (November to March) and the rainy season (April to October), interrupted by a short August break, reaching its peak in the months of June and September. Mean annual rainfall is about 1590 mm, with an average annual temperature of 27.5°C. Generally, the soil falls into the ferralitic group, characterized by deep soils with distinct profiles, higher natural fertility, and high water retaining capacity. Soils in Ijebu–ode city, like most cities around the world, have been transformed and are a mixture of original mineral soils, transported soils, organic materials, building materials (bricks, paint, concrete, metal), and wastes. Land uses in the city include residential housing, institutional, pavement, commercial land, and transportation, followed by industrial, agricultural, and uncultivated land (forest) use.

### Sampling design

Sampling locations within Ijebu-Ode city and environs were selected by a random sampling design using functional or land use activities, e.g. residential, industrial, commercial, forest, and transport regions, agricultural lands, urban vacant plots and institutional green spaces.^19^ The sampling locations were separated by distances ranging from 300 to 2000 m (*Figure 1*). Sampling locations were chosen with the aim of covering the entire city so that the number of samples would represent the different location types with different rates of land use activities in proportion to their ratio in the total sampling area.^20^ The sampling locations are described in Table 1.

**Table 1:** Geographical Coordinates and Land Use Types of Sampling Locations

S/No	Name	Longitude	Latitude	Land use
1	Ilamo (agricultural)	3.907333	6.850708	Agricultural (arable farm)
2	Oke-Owa fish farm	3.934295	6.808747	Aquaculture
3	Fish farm	3.884596	6.822484	Aquaculture
4	Oke-Eri mechanic village	3.931013	6.860782	Artisan (mechanic village)
5	Ayesan scrap market	3.919432	6.833113	Artisan (metal scrap market)
6	Mechanic-welder AGGS	3.902652	6.832796	Artisan (welder)
7	Oke-Eri Muslim cemetery	3.937362	6.859659	Cemetery
8	Imowo cattle market	3.93142	6.849716	Commercial (cattle market)
9	Oke-Aje Market	3.930535	6.818321	Commercial (market)
10	Sabo abattoir	3.900866	6.822985	Commercial (abattoir)
11	Refuse dumpsite	3.939614	6.791313	Dumpsite
12	Ijebu-Ode Forest Reserve	3.925118	6.862571	Forest
13	Frigoglass (Industrial)	3.897681	6.832445	Industrial
14	Tasued Ijagun	3.927455	6.783053	Institutional (Tasued University)
15	Ijebu-Ode grammar school	3.911273	6.82665	Institutional (Ijebu-Ode grammar school)
16	Odua High School Imoru	3.926878	6.798971	Institutional (Odua High School)
17	Ikangba housing estate	3.893013	6.838747	Residential (housing estate)
18	Sabo	3.897158	6.819533	Residential (housing estate)
19	Igbeba GRA	3.943888	6.827359	Residential (GRA)
20	Agoro	3.856961	6.842233	Suburb
21	Ibadan garage	3.921128	6.835544	Motor park
22	Egbe	3.892581	6.799683	Vehicle transport
23	Lagos garage	3.91017	6.810816	Motor park
24	Ijagun road	3.923383	6.807879	Vehicle transport

Abbreviations: AGGS, Anglican Girls Grammar School; GRA, Government Reserved Area

**Abbreviations**
*CR*: Cancer risk
*EF*: Enrichment factor
*HI*: Hazard index
*HQ*: Hazard quotient
*I_geo_*: Index of geo-accumulation
*RfD*: Reference doses
*SOM*: Soil organic matter
*USEPA*: United States Environmental Protection Agency

### Soil sampling and preparation

Twenty-four (24) representative plots of 20 m × 20 m were established across the different land-use types within the city and were sampled for soils once a month for three months (October – December 2015) in triplicates. Two hundred and sixteen (216) sub-samples comprised of seventy-two (72) composite soils (0–20 cm) were obtained by pooling and mixing five subsamples diagonally distributed in the plots to minimize local heterogeneity. One (1) kg of surface soils was collected from each site by hand digging using a soil auger. Nigeria has no record of background concentrations of heavy metals in urban soils, hence five soil samples were collected from the Ijebu-Ode Forest Reserve located near the city for comparison (reference values). In addition, three undisturbed 5-cmdiameter by 5-cm-deep cores were collected from each plot and used to measure bulk density. The geographical coordinates of samples sites were recorded using a handheld global positioning system (Garmin eTrex 10 GPS Unit).

Topsoil samples were collected and transferred to the laboratory in closed polyethylene bags. In the laboratory, the samples were air-dried at room temperature for several days and large rock, pebbles, and organic debris were removed before sieving. The samples were crushed and sieved through a mesh sieve to obtain fractions smaller than 2 mm, which were then ground into fine powder and stored in glass jars at 4°C for further analysis. The pH, soil organic matter (SOM), and particle size were determined by hydrometer method.^21^ Bulk density (g cm^−^3) was determined by core method.^22^ Heavy metal concentrations in each pre-dried soil samples were determined by acid digestion.^23^ The results of soil digestion by aqua regia was examined from three replicates and heavy metals (Cd, Cr, Cu, Mn, Ni, Pb, and Zn) in the filtrated solution were determined using atomic absorption spectrometry (AA-6300, Shimadzu, Japan). Background heavy metal concentrations were determined by taking soil samples from a forested area far from the sampling locations and devoid of any pollution sources.

### Quality control and quality assurance

In order to guarantee the accuracy of data, standard reference materials (certified reference materials BCR-141R, Brussels) were included in every batch of sample digestion and recovery study was carried out and analyzed as a part of the quality control protocol. Each sample was analyzed in triplicate and certified reference materials were tested after every 10 samples. The recovery studies of the metals varied between 99.3% and 100%. Samples were carefully handled to avoid contamination both on the field and in the laboratory to ensure reliability of results. The instrument was calibrated using standard solutions of the respective element. The calibration curves were linear within the concentration range. The detection limits (mgkg^−1^) of Cd, Cr, Cu, Mn, Ni, Pb, and Zn were 0.001, 0.005, 0.004, 0.001, 0.004, 0.001, and 0.034, respectively.

### Data analysis

Descriptive statistics including mean, median, maximum, minimum, standard deviation, and coefficient of variation were performed. Standard deviation and coefficient of variation were incorporated to represent the degree of dispersion distribution of different heavy metals and to indirectly indicate the activity of the selected elements in the examined environment.^24^ Correlation between heavy metals were determined using Pearson correlation analysis with the Statistical Package for the Social Sciences (SPSS) 17.0 software and Excel 2007 for Windows. The kriging interpolation of the contaminant concentrations was computed with the ArcGIS 10.2 software (Esri, Redlands, CA, USA).

### Pollution level analysis

The enrichment factor (EF) of the selected heavy metals in the surface soil was calculated. The EF represents the ratio of concentration of an element associated with anthropogenic pollution and a reference element in analyzed sample, relative to the corresponding ratio for the background concentrations. The reference elements normally used are: aluminum (Al), iron (Fe), lithium (Li), and Mn.^3^ Iron is regarded as a normalizing or reference element in this study.^25^ The EF was calculated using Equation 1.

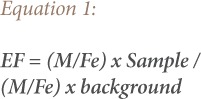
Where EF is the enrichment factor, ‘(M/Fe) sample’ is the ratio of metal and Fe concentration of the sample and ‘(M/Fe) background’ is the ratio of metals and Fe concentration of a background. The background concentrations of metals were taken from soils from an undisturbed area. The enrichment factor categories for the equation are outlined in the following tabulation.^25^

EF < 2
  Deficiently to minimal enrichment
2 ≤ EF < 5
  Moderate enrichment
5 ≤ EF < 20
  Significant enrichment
20 ≤ EF < 40
  Very high enrichment
EF ≥ 40
  Extremely high enrichment

An EF > 2 is considered an indication of heavy metal enrichment associated with anthropogenic pollution, while EF > 5 is assumed to imply significant enrichment.^25^

### Geo-accumulation index

The index of geo-accumulation (I_geo_) was computed.^26^ This index is considered to show both the impact of the geological process to natural background values and the influence of human activities on heavy metals pollution.^27^ It was calculated based on Equation 2.

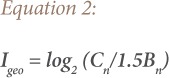
Where C_n_ is the concentration of the element in the tested soil, while B_n_ is the geochemical background value in the average shale of an element.^28^ The constant 1.5 compensates for natural fluctuations of a given metal and for minor anthropogenic impacts.^28^ The geo-accumulation index is calculated on the soil exchangeable fraction because it represents the real bioavailable fraction.^29^

The I_geo_ of Cr, Cu, Mn, Ni, Pb, and Zn were separately calculated and classified in the following tabulation 7.^26^

Class 0 (I_geo_ ≤ 0)
  Uncontaminated
Class 1 (0 < I_geo_ ≤1)
  Uncontaminated to moderately contaminated
Class 2 (1 < I_geo_ ≤2)
  Moderately contaminated
Class 3 (2 < I_geo_ ≤3)
  Moderately contaminated to heavily contaminated
Class 4 (3 < I_geo_ ≤4)
  Heavily contaminated
Class 5 (4 < I_geo_ ≤5)
  Heavily contaminated to extremely contaminated
Class 6 (I_geo_ ≥ 5)
  Extremely contaminated

### Health risk assessment model

Health risk assessment is a widely used method to assess and determine human exposure receptors to soil contamination due to land use.^30–31^ The intake doses occur via three main paths: ingestion, inhalation, and dermal contact.^31^ Exposures for both children and adults were calculated using Equations 3 through 5 below:

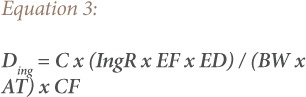


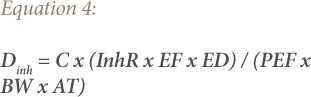


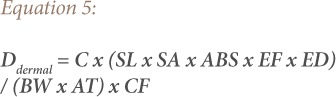
Where, D_ing_ is the daily dose via hand-to-mouth ingestion of soil substrate particles, D_inh_ is the daily dose via inhalation of re-suspended particles through the mouth and nose, and D_dermal_ is the daily dose via dermal absorption of trace elements in particles adhered to exposed skin. Furthermore, IngR is the ingestion rate (200 mg/d for children and 100 mg/d for adults), ED is exposure duration (6 y for children and 24 y for adults), EF is the exposure frequency (180 d/y), and BW is the average body weight (15 kg for children and 70 kg for adults).^10^,^30^,^31^ The InhR is the inhalation rate (7.6 m^3^/d for children and 20 m^3^/d for adults), PEF is the particle emission factor (1.36 × 109 m^3^ kg^−1^), and SA is the skin exposure area (2800 cm^2^ for children and 3300 cm^2^ for adults), SL for skin adherence factor (0.2 mg/cm^2^h for children and 0.7 mg/cm^2^h for adults).^31–34^ AT is average time (d) and AT is ED × 365 for non-carcinogenic risk and 70 × 365 for carcinogenic risk, and ABS is dermal absorption factor (0.001).^10^,^30^,^31^ CF is the unit conversion factor (mg kg^−1^) and is given by 1×10^−6^.

The exposure-point concentration, μg g^−1^ (C) was calculated as the upper limit of the 95% confidence limit for the mean (*Equation 6*).

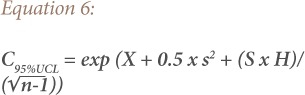
Where UCL is the upper confidence limit, X is the arithmetic mean of the log-transformed data, S represents the standard deviation of the log-transformed data, H is the H-statistic, and n is the number of samples.^35^

The exposure-point concentration, μg g^−1^ (C) in Equations 7–9 is an estimate of reasonable maximum exposure.^36^

### Health risk characterization

Assessment of each potentially toxic metal or risk characterization describes the likelihood and degree of chemical exposure at a site, the possible adverse health effects associated with such exposure, the quantification of carcinogenic or non-carcinogenic health risk, and a discussion of the uncertainties associated with the risk assessment. After calculating the dose received via each of the three paths, the doses of non-carcinogenic metals are divided by the corresponding reference doses (RfD) to yield a hazard quotient (HQ). The doses for carcinogens are multiplied by the corresponding slope factor to produce a cancer risk (CR). Hazard quotient and CR were calculated using Equations 7 and 8.

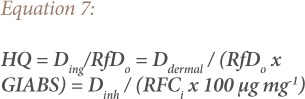


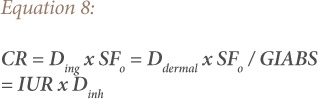
Where RfD_o_ is the oral reference dose (mg kg^−1^day^−1^), RfCi is the inhalation reference concentration (mgm^−3^), SF_o_ is the oral slope factor (mg kg^−1^day^−1^), GIABS is the gastrointestinal absorption factor, and IUR is the inhalation unit risk (mgm^−3^).^10^ Carcinogenic risk is the probability of an individual developing any type of cancer from lifetime exposure to carcinogenic hazards with the acceptable or tolerable risk for regulatory purposes ranging between 1 ×10^−6^ – 1 × 10^−4^.^3^,^6^ Risks above 1 × 10^−4^ are considered to be unacceptable, while risks below 1 × 10^−6^ are not thought to trigger any health effect.^10^,^14^ An HQ≤1 indicates no adverse health effects and HQ≥1 indicates likely adverse health effects.^21^ Furthermore, the hazard index (HI) (HI = Σ^i^_1_ HQ), which indicates the overall potential non-carcinogenic effects posed by more than one chemical, was calculated.^6^ A hazard index <1 indicates that there is no significant risk of non-carcinogenic effects and an HI>1 indicates that there is a chance of non-carcinogenic effects occurring.^10^ The HI, however, is not a direct estimate of the severity of the hazard, but simply an index to guide decision-making and further analysis. The cancer risk is expressed as a probability and is based on the cancer potency of the chemical, known as a cancer slope factor. The non-cancer hazard is expressed as the ratio of the chemical intake (dose) to a RfD. The chronic RfD represents an estimate (with uncertainty spanning perhaps an order of magnitude or greater) of a daily exposure level for the human population, including sensitive populations (e.g., children), that is likely to be without an appreciable risk of deleterious effects during a lifetime. The RfD and slope factor values of analyzed metals are listed in Table 2.^10^

**Table 2:** Reference Dose and Slope Factor of Heavy Metals ^a^

	RfDing (mg kg^−1^ day^−1^)	RfDinh (mg kg^−1^ day^−1^)	RfDder (mg kg^−1^ day^−1^)	SFinh (mg kg^−1^ day^−1^)
Cd	1.00 × 10^−3^	1.00 × 10^−3^	1.00 × 10^−5^	6.10
Cr	3.00 × 10^−3^	2.86 × 10^−5^	6.00 × 10^−5^	4.10 × 10
Cu	4.00 × 10^−2^	4.02 × 10^−2^	1.20 × 10^−2^	n.d.
Mn	1.40 × 10^−1^	5.00 × 10^−2^	1.40 × 10^−1^	n.d.
Ni	2.00 × 10^−2^	2.06 × 10^−2^	5.40 × 10^−3^	8.40 × 10^−1^
Pb	3.50 × 10^−3^	3.52 × 10^−3^	5.25 × 10^−4^	n.d.
Zn	3.00 × 10^−1^	3.00 × 10^−1^	6.00 × 10^−1^	n.d.

^a^USEPA^10^

Abbreviations: n.d., not determined; RfDing, reference dose via ingestion; RfDinh reference dose via inhalation; RfDder, reference dose via dermal contact; SFinh; slope factor via inhalation.

### Mapping soil metal concentrations

Total metal concentrations of Cd, Cr, Cu, Mn, Ni, Pb, and Zn in the soil samples were mapped using spatial interpolation, which is a geostatistical method.^37^,^38^ The technique of spatial interpolation predicts the values of an attribute at unsampled sites by incorporating information about the geographic positions of the sample points. This study employed the use of an interpolation known as ordinary kriging.

## Results

Soil pH ranged from 5.3 to 7.80. The highest pH was found in soils from Igbeba Government Reserved area (GRA), while the lowest pH was found in the Ijebu-Ode Forest Reserve. The soils in the study area were predominately sandy in texture (particle size > 50 μm) with a grainsize distribution of sand ranging from 68 to 89%. Soils from the roadsides, residential, and institutional areas contained a higher proportion of sand, while soils collected in the agricultural lands and forest contained higher clay contents. In general, clay content (< 2 μm) did not exceed 15% in any of the areas studied (*Table 3*), while silt content (2–50 μm) ranged from 3.47% in soils from the residential areas to 8.27% in the forest soils. Topsoil in the forest was classified as sandy clay loam.

**Table 3:** Soil Properties in the Sampling Locations

S/No	Location	Soil Properties					

		BD (g cm^−3^)	pH	SOM %	Sand %	Silt %	Clay %
1	Ilamo (Agricultural)	0.82	5.68	2.2	81	8.3	10.7
2	Oke-Owa Fish Farm	1.56	6.71	1.76	80	8	'12
3	Idomila Fish Farm	1.34	6.89	2.08	76	7.2	11
4	Oke-Eri Mechanic Village	1.66	6.34	0.95	82.1	7.9	10
5	Ayesan Scrap Market	1.7	6.56	0.83	82	5	13
6	Mechanic-Welder AGGS	1.69	6.49	0.64	80	5.2	14.8
7	Oke-Eri Muslim Cemetery	1.54	7.23	0.96	81	5.4	13.6
8	Imowo Cattle Market	1.78	7.56	0.84	83	7	10
9	Oke-Aje Market	1.82	7.68	0.76	79	8.1	12.9
10	Sabo Abattoir	1.77	7.34	0.97	81	6.5	12.5
11	Refuse Dumpsite	1.6	6.03	1.35	79	6	15
12	Ijebu-Ode Forest Reserve	0.56	5.32	2.54	80	8.27	12
13	Frigoglass (Industrial)	1.72	6.56	1.32	85	3.47	11,53
14	Tasued Ijagun (Institutional)	1.72	7.64	1.09	80.5	7	12.5
15	Ijebu-Ode Grammar School	1.65	6.96	0.87	88.4	7.3	4.3
16	Odua High School Imoru	1.53	7.23	0.9	85.8	6.5	10
17	Ikangba Housing Estate	1.78	7.8	0.65	81	6.7	12.7
18	Sabo (Suburb)	1.67	7.67	0.78	89	5.8	5.2
19	Igbeba GRA	1.81	7.82	0.94	87	6.4	6.6
20	Agoro (Suburb)	1.04	7.54	1.04	83	8.1	8.9
21	Ibadan Motor Garage	1.37	6.75	0.45	86	7,4	6.6
22	Egbe (Suburb)	1.22	6.69	0.53	87	6.3	6.7
23	Lagos Motor Garage	1.79	7.34	0.44	80	7.8	12.2
24	Ijagun Road (Transport)	1.65	7.2	0.61	79	7	14

Abbreviation: AGGS, Anglican Girls Grammar School; BD, bulk density, GRA, Government Reserved Area, SOM, soil organic matter

Bulk density ranged from 0.56 to 1.82 (g cm^−3^). The highest value for SOM was found in Ijebu-Ode Forest Reserve, while the lowest SOM value was found in Lagos Garage (motor park). Soil organic matter ranged from 0.44% to 2.54%.

### Concentrations of heavy metals in soils

Heavy metal contents of urban soils in Ijebu-Ode were spatially heterogeneous. Table 4 shows the concentrations of Cd, Cr, Cu, Mn, Ni, Pb, and Zn in urban surface soils collected from 24 different land use areas. The result showed that average abundance of the seven investigated heavy metals decreased in order of Zn > Pb > Mn > Cu > Cd > Ni > Cr. However, the highest concentration of all the metals was 239.04 mgkg^−1^ for Pb found in a dumpsite. However, the mean concentration of Pb (82.12 mgkg^−1^) was slightly lower than that of Zn (82.91 mgkg^−1^). Chromium exhibited the lowest concentrations at all sampling locations except for the dumpsite (8.02 mgkg^−1^), industrial (4.34 mgkg^−1^), and artisan workshops (3.21 mgkg^−1^ and 4.05 mgkg^−1^, respectively). Furthermore, the mean Ni concentration in the agricultural area was 4.02 mgkg^−1^. The mean concentration of Cu in the urban soil in Ijebu-Ode was 14.71 mgkg^−1^ with a maximum of 46.07 mgkg^−1^ measured in soils taken from the dumpsite (*Table 5*). The concentration of Cd in this study could not be attributed to industrial contaminants due to the absence of major industrial plants in the area, but substantial amounts of Cd are also released during weathering of ferralitic soil through oxidation and leaching.

**Table 4:** Concentrations of Heavy Metals in Urban Soils in Ijebu-Ode Across Sampling Locations (n=3)

S/No	Land use		Cd	Cr	Cu	Mn	Ni	Pb	Zn

			(mg kg^−1^)						
1	llamo (Agricultural)		1.06	1.03	2.11	10.07	4.02	12.08	34
2	Oke-Owa Fish Farm		0.88	0.78	2.35	18.02	3.41	67.23	45
3	Idomila Fish Farm		1.02	0.25	4.22	20.21	2.06	80.21	40
4	Oke-Eri Mechanic Village		6.79	3.87	20.01	78.34	20.13	203.7	118.3
5	Ayesan Scrap Market		6.09	4.05	45.02	80.17	18.04	177.2	156.09
6	Mechanic-Welder AGGS		1.66	3.21	13.58	92.01	13.02	154.03	148.17
7	Oke-Eri Muslim Cemetery		0.52	0.11	3.46	11.23	2.34	20.02	20.31
8	Imowo Cattle Market		2.04	2.03	23.45	20.01	2.08	112.04	67.32
9	Oke-Aje Market		2.27	2.76	21.04	22.56	3.75	125.08	50.12
10	Sabo Abattoir		1.06	2.23	12.39	12.39	2.33	76.23	44.16
11	Refuse Dumpsite		8.02	8.02	46.07	101.03	17.39	239.04	173.02
12	Ijebu-Ode Forest Reserve		0.23	0.12	1.09	4.02	1.02	10.03	19.05
13	Frigoglass (Industrial)		4.01	4.34	26.35	34.81	7.03	125.07	134.02
14	Tasued Ijagun (Institutional)		3.07	1.12	13.02	17.28	3.44	30.17	66.34
15	Ijebu-Ode Grammar School		3.35	0.86	14.18	12.34	4.01	12.04	65.18
16	Odua High School Imoru		2.11	1.03	14.02	19.65	3.07	24.05	49.25
17	Ikangba Housing Estate		2.23	1.01	11.09	11.28	3.11	10.28	34.2
18	Sabo (Suburb)		3.01	0.95	9.03	15.29	4.02	11.02	33.72
19	Igbeba GRA		1.67	0.78	4.23	20.12	3.02	8.22	34.19
20	Agoro (Suburb)		0.69	0.22	2.05	9.23	1.03	5.04	23.09
21	Ibadan Motor Garage		8.02	1.29	17.02	77.34	15.31	109.34	201.09
22	Egbe (Suburb)		0.21	0.62	10.23	23.95	5.03	45.02	45.82
23	Lagos Motor Garage		10.2	1.56	27.01	98.02	20.13	203.78	209.35
24	Ijagun Road (Transport)		3.44	1.02	10.05	56.09	10.02	110.02	178

(8)12	(100)300	(36)190	-		(35)210	(86)530	(140)720		
0.36–140	-	49–80	-		38–280	56–120	79–160		

^a^Value in parenthesis is the target value, while the other is the intervention value

Abbreviations: AGGS, Anglican Girls Grammar School; GRA, Government Reserved Area

**Table 5:** Summary of Total Heavy Metal Concentrations (mg/kg) of Urban Surface Soils in Ijebu-Ode

Heavy Metal	N	Minimum	Maximum	Mean	Median	SD	Kurtosis	Skewness	CV
Cd	24	0.21	10.20	3.07	2.17	2.77	0.73	1.26	90
Cr	24	0.11	8.02	1.80	1.03	1.82	4.84	2.00	101
Cu	24	1.09	46.07	14.71	12.71	12.23	1.64	1.31	83
Mn	24	4.02	101.03	36.06	20.07	32.49	−0.57	1.05	90
Ni	24	1.02	20.13	7.03	3.88	6.48	−0.32	1.12	92
Pb	24	5.04	239.04	82.12	71.73	73.05	−0.68	0.69	89
Zn	24	19.05	209.35	82.91	49.69	62.87	−0.78	0.88	76

Abbreviations: CV, coefficient of variation; SD, standard deviation.

**Figure 2:**
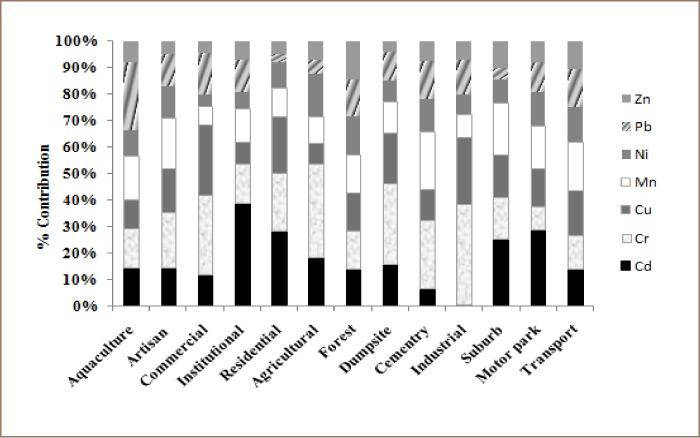
Percentage contributions of heavy metals across different land uses in the study area.

Forested and agricultural areas generally have low concentrations of heavy metal contaminants. Surface soils from dumpsite, artisan workshops, motor parks and transport routes have higher concentrations. Levels of heavy metal contamination across different urban land use types followed a pattern in which heavy metal concentrations were highest in dumpsite and high-traffic areas, followed by residential and commercial areas (*Figure 2*).

Although there are variations in concentrations of heavy metals in soils from the different land uses, all metals exhibit strong positive correlations with each other at 0.05 level of significance (*Table 6*).

**Table 6:** Pearson's Correlation Coefficients Among Heavy Metals in Soil Across Land Uses

	Cd	Cr	Cu	Mn	Ni	Pb	Zn
Cd	1.000						
Cr	0.654^*^	1.000					
Cu	0.833^**^	0.938^**^	1.000				
Mn	0.787^*^	0.723^*^	0.780^*^	1.000			
Ni	0.859^**^	0.692^*^	0.789^*^	0.974^**^	1.000		
Pb	0.790^**^	0.857^**^	0.914^**^	0.920^**^	0.896^**^	1.000	
Zn	0.858^**^	0.679^*^	0.799^**^	0.931^**^	0.942^**^	0.897^**^	1.000

^*^Correlation is significant at the 0.01 level (2-tailed)

^**^Correlation is significant at the 0.05 level (2-tailed)

Degree of freedom=N-2

### Assessment of contamination

The enrichment factors were calculated for the different land use categories based on mean heavy metal concentrations in the different sampling sites. The land use classifications are presented in Table 7.

**Table 7:** Land Use Classifications

Land use type	Sampling sites
Aquaculture	Oke-Owa and Idomila Fish Farms
Artisan	Oke-Eri Mechanic Village, Ayesan Scrap Market, and Mechanic-Welder AGGS
Commercial	Imowo Cattle Market and Oke-Aje Market
Institutional	Ijebu-Ode Grammar School and Odua High School, Imoru
Residential	Ikangba Housing Estate and Igbeba GRA
Agricultural	Ilamo
Dumpsite	refuse dumpsite
Cemetery	cemetery
Industrial	Frigoglass
Suburb	Sabo, Agoro and Egbe
Motor park	Ibadan and Lagos garages
Vehicle Transport	Ijagun Road

Enrichment factors (*Table 8*) revealed significant enrichment of Cd in the dumpsite (5.173), motor park (6.928), and institutional (11.221) soils. Chromium, Cu, and Mn were also significantly enriched (5 ≤ EF < 20) in soils from artisan workshops with enrichment factors of 6.675, 5.190, and 6.000, respectively.

**Table 8:** Enrichment Factor of Heavy Metals Across Land Use Types

Land use type^*^	Cd	Cr	Cu	Mn	Ni	Pb	Zn
Aquaculture	3.436	3.570	2.507	3.955	2.230	6.114	1.856
Artisan	4.550	6.675	5.190	6.000	3.612	3.838	1.596
Commercial	1.916	4.800	4.282	1.122	0.656	2.563	0.696
Institutional	11.221	4.204	2.325	3.575	1.737	3.525	2.033
Residential	2.624	1.994	1.951	1.014	0.869	0.257	0.468
Agricultural	4.057	7.557	1.704	2.205	3.470	1.060	1.571
Dumpsite	5.173	9.915	6.270	3.728	2.529	3.536	1.347
Cemetery	3.480	12.899	6.008	11.037	6.155	7.405	3.751
Industrial	0.217	9.477	6.335	2.269	1.806	3.268	1.844
Suburb	1.447	0.884	0.907	1.107	0.487	0.242	0.584
Motor park	6.928	2.077	3.532	3.815	3.038	2.730	1.884
Vehicle Transport	1.708	1.471	2.003	2.144	1.588	1.664	1.265

^*^Land use classifications are described in Table 7

Copper was moderately enriched (2 ≤ EF < 5) across the majority of land use types investigated in this study except for agricultural, residential, and suburb areas.

### Geo-accumulation index

The average I_geo_ values of Mn, Pb, and Zn were 6.322, 8.394, and 9.431, respectively, indicating that the urban soils in Ijebu-Ode were highly contaminated by these metals (*Table 9*). Cadmium and Cr had I_geo_ values of 0 < I_geo_ ≤ 1, indicating non-contamination to moderate contamination.

**Table 9:** Heavy Metal Geo-Accumulation Index Values Across Study Area

Geo-accumulation index							

	Cd	Cr	Cu	Mn	Ni	Pb	Zn
Aquaculture	−2.779	−4.601	1.255	5.679	0.895	8.945	9.076
Artisan	−0.428	−1.752	4.251	7.806	3.536	10.220	10.805
Commercial	−1.865	−2.417	3.784	5.618	0.887	9.448	9.418
Institutional	−3.738	−7.031	−1.520	2.867	−2.132	5.485	6.542
Residential	−1.502	−3.775	2.560	5.382	1.202	6.040	8.756
Agricultural	−2.621	−3.601	0.617	4.754	1.451	6.336	8.754
Dumpsite	0.298	−0.640	5.065	8.081	3.564	10.642	11.102
Cemetery	−1.974	−1.961	3.303	7.946	3.146	10.008	10.878
Industrial	−0.702	−1.526	4.259	6.544	2.257	9.708	10.733
Suburb	−3.241	−5.828	0.779	5.127	−0.702	4.910	7.126
Motor park	0.482	−3.133	5.963	8.834	5.385	10.059	10.530
Vehicle Transport	−1.837	−3.930	4.677	7.228	2.154	8.932	9.457

^*^Land use classifications are described in Table 7

### Non-carcinogenic risk assessment

**Figure 3:**
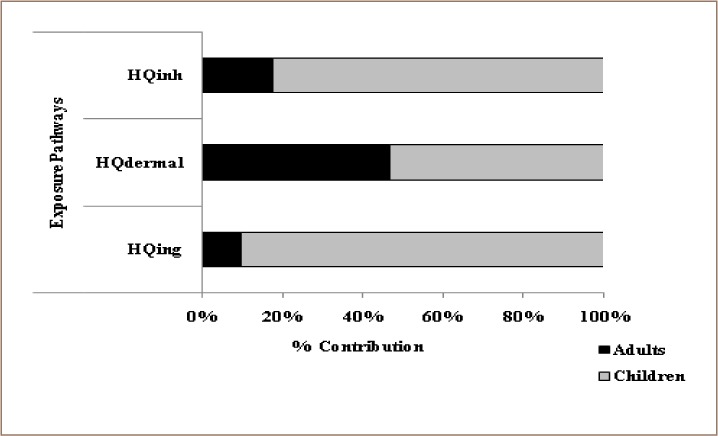
Percentage of non-carcinogenic risk distribution of different exposure pathways for adults and children in Ijebu-ode

The exposure scenario for both adults and children in the contaminated areas was examined. The HQs and HI for the heavy metals (Cd, Cr, Cu, Mn, Ni, Pb, and Zn) were investigated for both adults and children for the three exposure pathways (inhalation, ingestion, and dermal contact) in the study area. The daily doses of Cu, Ni, Pb, and Zn by ingestion and inhalation were 3–6 orders of magnitude higher than dermal contact (*Supplemental Material*). The study also revealed that the 95% upper confidence limit values of the total exposure doses of Mn, Pb, and Zn were several orders (13–265) of magnitude higher than for Cd, Cr, Cu, and Ni. Ingestion is the main exposure pathway that posed the highest risk level. For adults, the highest level of risk came via dermal contact, while HQing values for adults were about 10 orders of magnitude lower compared to children. However, HI values were 3.42 for Mn in children and 7.88 for Pb in adults, which exceeded the safe limit (=1). Figure 3 shows the percentage non-carcinogenic risk distribution of the different exposure pathways for both children and adults in soils across the study area.

**Figure 4:**
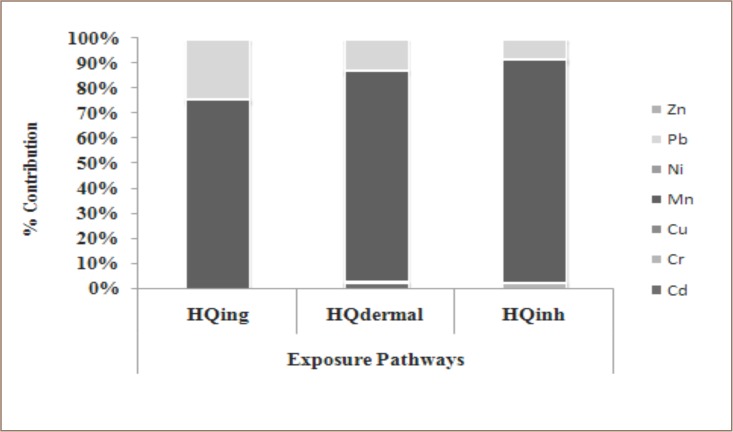
Percentage contributions of heavy metals to different exposure pathways

HQ_inh_ shows the hazard quotient for inhalation, HQ_ing_ is the hazard quotient for ingestion and HQ_dermal_ is the hazard quotient for dermal contact. The graph shows that ingestion and inhalation had the highest percentages of hazard quotient in children (90% and 85% respectively), while hazard quotient for dermal contact was higher in adults. The hazard index for all studied metals was ranked in the order of Ni < Cu < Cr < Zn < Cd < Pb < Mn for adults and Cr < Ni < Cu < Zn < Cd < Pb < Mn for children. In the present study, Mn, Pb, Cd, and Zn were the main contributors to health risks posed by soil contaminated by heavy metals exposure for both adults and children. For each metal, the different exposure routes and investigated heavy metals showed varying contributions to the HQs. In both adults and children, Mn contributed the most to all of the HQs (*Figure 4*). The percentage contribution of Mn from different exposure pathways was 75%, 84%, and 89% for HQ_ing_, HQ_dermal_, and HQ_inh_, respectively.

### Carcinogenic risk assessment

The CR for both adults and children exposed to contaminated soils indicates that Pb, Zn, and Cu had the highest cancer risks for both adults and children (*Supplemental Material*).

Furthermore, Cu, Mn, Pb, and Zn were higher than the acceptable range of 1 × 10^−6^ – 1 × 10^−4^ for children (1.50 × 10^−3^ – 2.71 × 10^−2^), and Mn, Pb, and Zn were higher than the acceptable range of 1 × 10^−6^ – 1 × 10^−4^ for adults (7.89 × 10^−4^ – 2.97 × 10^−3^). Cancer risk values indicated that children had the highest potential cancer risk. The risk of cancer among the heavy metals decreased in the order Pb>Zn>Mn>Cu>Ni>Cd>Cr.

### Spatial mapping of heavy metal distributions in topsoil

Variogram models were used to analyze spatial patterns and ordinary kriging was used for mapping predictions of the concentrations of the eight heavy metals (Cd, Cr, Cu, Fe, Mn, Ni, Pb, and Zn). Mapping showed spatial patterns and high variation in the concentrations of the investigated heavy metals (*Figure 4*).

## Discussion

Sandy soils are often acidic, which is shown by the low pH of some of the surface soils investigated in Ijebu-ode. Soil pH increased from low (acidic) pH of soils sampled from forest and agricultural lands to neutral pH in residential and institutional areas. Soil pH ranging from 5.1 to 7.6 in residential soils was observed in Ibadan, Nigeria, similar to the results of the present study.^40^ Soil samples collected from roadsides and a courtyard in Hong Kong, however, recorded a higher (and occasionally basic) pH.^41^ Soils from the Ijebu-Ode Forest Reserve had the lowest bulk density, while the highest bulk density was found in soil taken from Oke-Aje Market, a commercial area. Studies of urban surface soils in Baltimore, Hong Kon,g and Moscow reported higher bulk densities in the range of 1.4 and 1.7 Mg m^−3^, especially for residential soils.^41^ This is due to intense human disturbance such as trampling.^2^,^5^ High bulk densities are an indicator of soil compaction, which impacts soil porosity, water capacity, root growth, and movement of air and water through soil. Soil organic matter contents varied widely across the different sampling locations, with the highest proportion (2.54%) detected in forest soil, which is relatively undisturbed. Ijebu-Ode Forest Reserve had the lowest pH and bulk density, which indicates that the area has not been polluted by anthropogenic activities.

Concentrations of Pb and Zn in the urban soils in some areas exceeded the United States Environmental Protection Agency (USEPA) ecological-soil screening levels (ECOSSL). 42 The mean concentration of Pb (82.12 mgkg^−1^) in urban soils in Ijebu-Ode was 3-fold higher than the concentration of 25.36 mgkg^−1^ reported in urban soils in Beijing and 22.17 mgkg^−1^ in Fuxin City, China.^8^,^38^ Table 2 shows the summary statistics of heavy metal concentrations in urban soils in Ijebu-Ode across all examined land uses. The concentration of Zn (82.91 mgkg^−1^) in this study was slightly higher than that of 80.28 mgkg^−1^ reported in urban soils in Beijing and much higher than the 54.21 mgkg^−1^ concentration found in Fuxin City, China.^8^,^38^ High Pb concentrations in urban soils could also be explained by traffic emission and use of leaded paints in artisan workshops. In addition, Zn has been reported to have high mobility and is likely to migrate down through the soil profile. Chromium is regarded as a pollutant from industrial activities, but it is also a metal indicating geogenic load, which could be released from parent rocks through rapid weathering.^43^ The mean concentration of Cr in this study was much lower than the 51.08 mgkg^−1^ in Fuxin City, China, probably because Ijebu-Ode is not as industrialized as Fuxin City.^38^

The maximum mean concentration of Cu in this study was higher than the 28.82 mgkg^−1^ and 31.63 mgkg^−1^ measured in industrial and roadside soils in Beijing metropolitan region, China.^11^ The mean Cu concentration for urban soils in Ijebu-ode (14.71 mgkg^−1^) was, however, lower than the 30.17 mgkg^−1^ level recorded for residential areas in Beijing.^11^ Copper is a metal with very low mobility and studies have shown that it tends to accumulate in topsoil.^3^,^43^ Major sources of Cu include the deterioration of some mechanical parts (e.g. lining in automobile brakes) in vehicles over time. The maximum concentration of Cd (10.2 mgkg^−1^) found in the soils was at a motor park in the city (*Table 2). *However, this value was lower than the Dutch soil guideline of 12 mgkg^−1^.^39^ Cadmium concentrations in the agricultural soil samples was 1.06 mgkg^−1^, which is lower than the USEPA ecological limit.^42^ However, with increasing human activities, Cd can readily enter the food chain via soil-crop systems, leading to potential food safety and human health risks.^8^ Compared with similar studies of urban soils in other cities around the world, the mean concentration of Cd in the present study (3.07± 2.77 mgkg^−1^) was higher than the level of 0.19± 0.11 mgkg^−1^ reported in in Fuxin City, China. Moreover, Ogunkunle and Fatoba reported Cd concentrations in the range of 8.4± 19.78 mgkg^−1^ in Ibadan, Nigeria.^43^ Conversely, the maximum concentration of Ni was lower than international standards, including the USEPA ECO-SSL limit of 38–280 mgkg^−1^ and the Dutch target values of 35 mgkg^−1^ and soil remediation intervention value of 210 mgkg^−1^.^39^,^42^

The target values are underpinned by an environmental risk analysis wherever possible and apply to individual substances, and the soil remediation intervention values indicate when the functional properties of the soil for humans, plants and animals are seriously impaired or threatened. They are representative of the level of contamination above which a serious case of soil contamination is deemed to exist.^39^ Furthermore, the mean Ni concentration in the agricultural area was 4.02 mgkg^−1^, compared to 24.57 mgkg^−1^ recorded in farmland in Fuxin, northeastern China.^38^ Among the heavy metals investigated in the urban soils in Ijebu-Ode, mean concentrations of Cd, Cu, and Ni were within the USEPA ECO-SSL for plants, soil, invertebrates, and mammalian wildlife.^42^ Accumulation of Cd, Cu, and Zn in urban soils along transport routes could be attributed to the abrasion of tires and vehicle parts.^11^,^44–46^

The mean Mn concentration in this study was 36.06 mgkg^−1^, with a maximum of 101.03 mgkg^−1^ found in the dumpsite soil. The concentration of Mn was also higher in soils at the motor park and artisan workshop, which were, however, lower than the levels of 708, 721 683, 677, and 646 mgkg^−1^ recorded for industrial, roadside, residential, institutional, and forest soils, respectively, in Beijing, China.^11^ Soils from transport routes or roadsides and residential areas have high concentrations of heavy metals because they were frequently disturbed by human activities. This finding was consistent with similar earlier studies.^5^,^11^ Another factor that may increase concentrations of the heavy metals is the low pH of some of the surface soils. Low pH in the soil may make previously accumulated heavy metals become rapidly soluble, thus increasing their toxic effects. With increasing anthropogenic activities in the study area, pollution of urban soil by heavy metals poses a threat to human health.^42^,^47–49^ Variation in metal concentrations observed in urban soils in the study area is a common phenomenon worldwide due to different patterns of contamination, land use, population density, socioeconomic development, environmental regulations, and local climate conditions.^45^,^49^

Zinc was the least enriched of the heavy metals in urban soils in Ijebu-Ode (*Table 5*), with EF values generally <2, except in the institutional (2.033) and cemetery soils (3.751). Enrichment factor in metals is an indicator of the presence and intensity of anthropogenic contaminant deposition on surface soil.^29^ Across the different locations, residential soils had the least enrichment with heavy metals (*Table 5*). Iron was measured in the soils but not included in Table 2 because it is often the most abundant in soil. However, the mean concentration of Fe in the study area was 342.32±163.72 mg/kg, showing spatial variation.

Contamination levels of heavy metals in urban soils in the study area were also assessed using I_geo_ because it is very important to distinguish between the natural background values and anthropogenic inputs.^33^ The average I_geo_ value for Zn (9.431) was the highest among all the metals investigated, while Mn and Pb had average I_geo_ values of 6.322 and 8.394 respectively, suggesting extreme contamination of the soil. These metals pose major problems in soil, especially at concentrations above certain thresholds.^13^ Long-term inputs of heavy metals could result in decreased buffering capacity of soil, threatening the ecological environment. This study employed the use of an interpolation method known as ordinary kriging.^17^ Heavy metals exhibited high spatial variability in the study area as shown in Figure 5. The mapping showed that the concentration of heavy metals is high in areas such as dumpsites and artisan workshops.

### Health risk assessment

The release of heavy metals into soil can pose a significant potential environmental and human health threats to people living around such environments.^17^,^29^ The maximum exposure doses for Zn in both adults and children are 3.00E-01, and 6.00E-02, respectively. Ingestion was found to be the main exposure pathway with the highest risk level, especially for children, as corroborated by similar studies.^18^,^38^ The non-cancer risk posed by dermal contact was equally high in both adults and children in the present study, unlike other studies.^18^,^36^ Hazard index values for most of the heavy metals investigated in this study were within the safe level (=1), suggesting minimal non-carcinogenic risk to children and adults from exposure to the contaminated soil in the study area. Hazard index values higher than 1 indicate that there is a chance of non-carcinogenic effects occurring due to exposure to heavy metals in the soil.^50^ Cancer risks for Cu, Mn, Pb, and Zn were not within the acceptable range of 1 × 10^−6^ – 1 × 10^−4^ for both adults and children, and children had the highest potential cancer risk.

## Conclusions

The present study integrated land use information to create realistic estimates of potential risks of heavy metals to human health through exposure to urban soil contamination in Ijebu-Ode, Nigeria. The low soil pH observed in some parts of the study area could enhance the mobility of these heavy metals. The majority of the land use areas were substantially contaminated by the heavy metals investigated, except for Ni. Increasing anthropogenic activities in the study area have influenced the accumulation of heavy metals such as Cd, Cu, Mn, Pb, and Zn in the urban soils. Lead and Zn concentrations in the urban soils in areas such as artisan workshops and dumpsites exceeded international threshold limits. High Pb concentrations in the urban soils could also be due to increasing traffic emissions and use of leaded paints in artisan workshops. Mapping of the study area showed spatial variation in the distribution pattern of heavy metal pollution in urban soils. Soils in the study area can be categorized as moderately contaminated. The noncancer risk posed by dermal contact was equally high in both adults and children, while the cancer risks for Cu, Mn, Pb, and Zn were higher than the acceptable range of 1 × 10^−6^ – 1 × 10^−4^, with children more susceptible than adults. There is the need for proper control and monitoring of urban activities that release heavy metals into the soil in order to safeguard human health.
