# Supplementary material for: Spatial Distribution and Health Risk Assessment of Soil Pollution by Heavy Metals in Ijebu-Ode, Nigeria
Source: J Health Pollut. 2019 May 20;9(22):190601. doi: 10.5696/2156-9614-9.22.190601 (PMC6555248; doi:10.5696/2156-9614-9.22.190601)
Supplement: Supplementary file 1 [file Adedeji_Supplemental_Material.docx]

Supplemental Material

Exposure Dose, Hazard Quotient and Cancer Risk Across Elements and Exposure Pathways (mg/kg-d)

| Element | C (95%UCL) | RfDing | RfDinh | RfDinh | HQ ingestion | | HQ inhalation | | HQ dermal | | HI | | CR | |
| --- | --- | --- | --- | --- | --- | --- | --- | --- | --- | --- | --- | --- | --- | --- |
|  |  |  |  |  | Adults | Children | Adults | Children | Adults | Children | Adults | Children | Adults | Children |
| Cd | 15.51 | 1.00E-03 | 1.00E-03 | 1.00E-05 | 1.09E-02 | 1.02E-01 | 1.61E-06 | 7.5E-06 | 2.52E-02 | 2.86E-02 | 3.62E-02 | 1.31E-01 | 1.12E-05 | 1.02E-04 |
| Cr | 9.32 | 3.00E-03 | 2.86E-05 | 6.00E-05 | 2.19E-03 | 2.04E-02 | 3.38E-05 | 0.000158 | 2.53E-03 | 2.86E-03 | 4.75E-03 | 2.34E-02 | 6.72E-06 | 6.15E-05 |
| Cu | 238.06 | 4.00E-02 | 4.02E-02 | 1.20E-02 | 4.19E-03 | 3.91E-02 | 6.14E-07 | 2.86E-06 | 3.23E-04 | 3.65E-04 | 4.52E-03 | 3.95E-02 | 1.72E-04 | 1.57E-03 |
| Mn | 1095.01 | 3.00E-04 | 8.57E-05 | 2.10E-05 | 2.57E+00 | 2.40E+01 | 1.32E-03 | 6.18E-03 | 8.49E-01 | 9.60E-01 | 3.42E+00 | 2.50E+01 | 7.89E-04 | 7.22E-03 |
| Ni | 74.92 | 2.00E-02 | 2.06E-02 | 5.40E-03 | 2.64E-03 | 2.46E-02 | 3.77E-07 | 1.76E-06 | 2.26E-04 | 2.55E-04 | 2.87E-03 | 2.49E-02 | 5.40E-05 | 4.94E-04 |
| Pb | 4115.79 | 3.50E-03 | 3.52E-03 | 5.25E-04 | 8.28E-01 | 7.73E+00 | 1.21E-04 | 5.65E-04 | 1.28E-01 | 1.44E-01 | 9.56E-01 | 7.88E+00 | 2.97E-03 | 2.71E-02 |
| Zn | 3195.53 | 3.00E-01 | 3.00E-01 | 6.00E-02 | 7.50E-03 | 7.00E-02 | 1.1E-06 | 5.15E-06 | 8.67E-04 | 9.81E-04 | 8.37E-03 | 7.10E-02 | 2.30E-03 | 2.11E-02 |

Abbreviations: C, exposure-point concentration; UCL, upper confidence limit; RfDing, reference dose via ingestion; RfDinh reference dose via inhalation; RfDder, reference dose via dermal contact.
